# Editorial of Special Issue “Protective and Detrimental Role of Heme Oxygenase-1”: 2021

**DOI:** 10.3390/ijms24054386

**Published:** 2023-02-23

**Authors:** Valeria Sorrenti

**Affiliations:** Department of Drug and Health Sciences, Biochemistry Section, University of Catania, 95125 Catania, Italy; sorrenti@unict.it; Tel.: +39-0947384115

The Special Issue “Protective and detrimental role of heme oxygenase-1”(2021), in the *International Journal of Molecular Sciences*, includes original research papers and reviews aiming to understand the protective or detrimental role of HO-1 and the signaling pathway involved.

Heme oxygenase (HO) is the enzyme that catalyzes heme degradation with the simultaneous release of carbon monoxide (CO), ferrous iron (Fe^2+^), and biliverdin, which is then reduced to bilirubin. Two main isoforms of heme oxygenase are present in humans: HO-1 (inducible) and HO-2 (constitutive). HO-1 may be induced in many organs and tissues in various stress-related conditions in order to obtain a cytoprotective effect. Due to its cytoprotective effects, HO-1 upregulation may be useful in many stress-related diseases. However, HO-1 overexpression may contribute to tissue injury under certain unfavorable conditions, such as neurodegeneration and carcinogenesis. Therefore, original research papers and reviews aiming to identify natural molecules or new synthetic compounds able to modulate HO-1 activity/expression will assist the use of HO-1 as a potential therapeutic target for the amelioration of various diseases.

The modulation of heme content by heme oxygenase activity can affect other heme proteins, such as Indoleamine 2,3-dioxygenase 1 (IDO1), a heme enzyme considered attractive for its significant function in cancer immunotherapy. Yan et al. performed a systematic study of analogs of SAR405838, a spiro-oxindole skeleton compound able to selectively inhibit murine double minute 2 (MDM2) protein, and also evaluated its capacity to inhibit IDO1 activity. The authors, by screening all synthesized compounds with a structural similarity to tryptophan, demonstrated that one synthesized compound exhibited the highest IDO1 inhibitory activity and was found to have the most effective inhibition of IDO1 in MCF-7 cancer cells without toxic effects. Therefore, this study provides valuable insights into the screening of more potent IDO1 inhibitors for cancer immunotherapy [1]. 

Recently, particular research interest has been focused on identifying the role played by heme oxygenase in the ferroptosis process. The term ferroptosis refers to a peculiar type of programmed cell death (PCD) mainly characterized by extensive iron-dependent lipid peroxidation. Ferroptosis has been suggested as a potential new strategy for the treatment of several cancers, including breast cancer (BC). In particular, among the BC subtypes, triple-negative breast cancer (TNBC) is considered the most aggressive, and conventional drugs fail to provide long-term efficacy. In this context, the purpose of Consoli et al.’s study was to investigate the mechanism of ferroptosis in breast cancer cell lines and reveal the significance of heme oxygenase (HO) modulation in the process, providing new biochemical approaches. HO’s effect on BC was evaluated using MTT tests, gene silencing, Western blot analysis, and measurement of reactive oxygen species (ROS), glutathione (GSH) and lipid hydroperoxide (LOOH) levels. In order to assess the implications of HO effects, different approaches were exploited, using two distinct HO-1 inducers (hemin and curcumin), a well-known HO inhibitor (SnMP) and a selective HO-2 inhibitor. The data obtained showed HO’s contribution to the onset of ferroptosis; in particular, HO-1 induction seemed to accelerate the process. Moreover, the results obtained by Consoli et al. suggest a potential role of HO-2 in erastin-induced ferroptosis. In view of the above, HO modulation in ferroptosis can offer a novel approach for breast cancer treatment [2].

It has been reported that HO-1 induction may ameliorate various inflammatory diseases. However, few studies have investigated the role of HO-1 in cholestatic liver diseases, which can progress to end-stage liver disease and reduce patients’ quality of life. To this end, Kim et al. examined whether pharmacological induction of HO-1 by cobalt protoporphyrin (CoPP) ameliorates cholestatic liver injury. A murine model of 3,5-diethoxycarbonyl-1,4-dihydrocollidine (DDC) diet feeding was used. The administration of CoPP ameliorated liver damage and cholestasis with HO-1 upregulation in DDC-diet-fed mice. Induction of HO-1 by CoPP suppressed the DDC-diet-induced oxidative stress and hepatocyte apoptosis. In addition, CoPP attenuated cytokine production and inflammatory cell infiltration. Furthermore, the deposition of the extracellular matrix and expression of fibrosis-related genes after DDC feeding were also decreased by CoPP. HO-1 induction decreased the number of myofibroblasts and inhibited the transforming growth factor-β pathway. Altogether, these data suggest that the pharmacological induction of HO-1 ameliorates cholestatic liver disease by suppressing oxidative stress, hepatocyte apoptosis, and inflammation [3].

Choi et al.’s review discusses the dual roles of HO-1 and its metabolites in various neurovascular diseases, including age-related macular degeneration, ischemia-reperfusion injury, traumatic brain injury, Gilbert’s syndrome, and AD. Heme oxygenase (HO) has both beneficial and detrimental effects via its metabolites, including carbon monoxide (CO), biliverdin or bilirubin, and ferrous iron. In brains injured by trauma, ischemia reperfusion, or Alzheimer’s disease (AD), the long-term expression of HO-1 can be detected, which can lead to cytotoxic ferroptosis via iron accumulation. In contrast, the transient induction of HO-1 in the peri-injured region may have regenerative potential (e.g., angiogenesis, neurogenesis, and mitochondrial biogenesis) and neurovascular protective effects through the CO-mediated signaling pathway, the antioxidant properties of bilirubin, and iron-mediated ferritin synthesis [4].

Today, natural products play increasingly important roles, especially in early drug development [5,6]. Natural molecules or new synthetic compounds may be able to modulate HO-1 activity. Natural molecules can be extracted from vegetable matrices or can be obtained by the chemical synthesis of different enzymes. Deng et al.’s review summarizes the most important enzymes, such as flavin adenine dinucleotide (FAD)-dependent monooxygenases (FMOs), which are involved in the synthesis of many natural products. The authors introduce the mechanisms for different FMOs and summarize the difference between FMOs and cytochrome P450 (CYP450) mono-oxygenases, emphasizing the advantages of FMOs and their specificity for substrates. Finally, the authors present examples of FMO-catalyzed synthesis of natural products [7].

Overall, the contributions published in this Special Issue highlight the protective and detrimental role of HO-1 and the signaling pathway involved. Natural molecules or new synthetic compounds able to modulate HO-1 activity/expression may represent a therapeutic strategy against various diseases. In particular, HO-1 may represent a potential target for the amelioration of inflammatory diseases or cancer.
